# Developmental pattern of the cortical topology in high‐functioning individuals with autism spectrum disorder

**DOI:** 10.1002/hbm.25251

**Published:** 2020-10-21

**Authors:** Weihao Zheng, Zhiyong Zhao, Zhe Zhang, Tingting Liu, Yi Zhang, Jin Fan, Dan Wu

**Affiliations:** ^1^ Key Laboratory for Biomedical Engineering of Ministry of Education, College of Biomedical Engineering and Instrument Science Zhejiang University Hangzhou People's Republic of China; ^2^ Department of Psychology Queens College, The City University of New York New York New York USA

**Keywords:** autism spectrum disorder (ASD), graph theoretical analysis, networks of structural covariance, topological developmental pattern

## Abstract

A number of studies have indicated alterations of brain morphology in individuals with autism spectrum disorder (ASD); however, how ASD influences the topological organization of the brain cortex at different developmental stages is not yet well characterized. In this study, we used structural images of 492 high‐functioning participants in the Autism Brain Imaging Data Exchange database acquired from 17 international imaging centers, including 75 autistic children (age 7–11 years), 91 adolescents with ASD (age 12–17 years), and 80 young adults with ASD (age 18–29 years), and 246 typically developing controls (TDCs) that were age, gender, handedness, and full‐scale IQ matched. Cortical thickness (CT) and surface area (SA) were extracted and the covariance between cortical regions across participants were treated as a network to examine developmental patterns of the cortical topological organization at different stages. A center‐paired resampling strategy was developed to control the center bias during the permutation test. Compared with the TDCs, network of SA (but not CT) of individuals with ASD showed reduced small‐worldness in childhood, and the network hubs were reorganized in the adulthood such that hubs inclined to connect with nonhub nodes and demonstrated more dispersed spatial distribution. Furthermore, the SA network of the ASD cohort exhibited increased segregation of the inferior parietal lobule and prefrontal cortex, and insular‐opercular cortex in all three age groups, resulting in the emergence of two unique modules in the autistic brain. Our findings suggested that individuals with ASD may undergo remarkable remodeling of the cortical topology from childhood to adulthood, which may be associated with the altered social and cognitive functions in ASD.

## Graphical abstract

This article investigated the developmental trajectory of cortical topology in individuals with autism spectrum disorder (ASD) from childhood to adulthood using T1 images acquired from 17 international centers. We found that individuals with ASD were accompanied with reduced small‐worldness in the network of surface area (but not cortical thickness) in the childhood, followed by dispersed hub distribution and decreased network assortativity in adulthood. In addition, there was an induced cross‐age modular reorganization that reorganized insular‐opercular cortex and superior temporal cortex into a new module. These changes indicated reduced robustness of the cortical network, and impaired integration of multiple cognitive functions, leading to the dysfunction in autistic brain across life span.

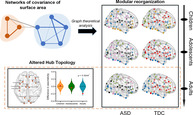

## 1 INTRODUCTION

Autism spectrum disorder (ASD) is a pervasive neurodevelopmental disorder characterized by impaired social–emotional functioning and related behavior throughout the lifespan, including deficits in social communication, restricted interest, and repetitive and stereotyped behaviors (Association, 2013; Baron‐Cohen, 2000). Studies have indicated an abnormal cerebral overgrowth in young children with ASD (age of 2–4 years) relative to typically developing children (Carper & Courchesne, 2005; Courchesne, 2002; Courchesne et al., 2001; Courchesne, Carper, & Akshoomoff, 2003; Hazlett et al., 2005), which was probably driven by the expansion of cortical surface area (SA) (Hazlett et al., 2011). Moreover, SA of 6‐12‐month old infants were able to predict the diagnosis of autism at 24 months (Hazlett et al., 2017). These findings suggested that the early structural changes in the brain cortex may be early signs of autistic symptoms. Towards later developmental stages, reduced anatomical abnormalities were reported in some studies (Courchesne et al., 2001; Courchesne, Campbell, & Solso, 2011; Lange et al., 2015; Redcay & Courchesne, 2005), though adolescents and adults with ASD maintained autistic symptoms. However, other studies showed that the gray matter (GM) may still undergo evident changes in the adults with ASD, especially in the amygdala and frontal brain regions (Courchesne et al., 2011; Ecker, Ginestet, et al., 2013; Eilam‐Stock, Wu, Spagna, Egan, & Fan, 2016; Freitag et al., 2009; Hazlett, Poe, Gerig, Smith, & Piven, 2006). These findings primarily concerned about the changes in GM morphology but did not address how the brain morphological networks were altered in terms of connectivity, hub topology (the spatial distribution of important brain regions and their connective pattern), and modularity (measures the decomposability of a network into several sparsely interconnected communities) at different stages of development.

The alterations in autistic brain have recently been investigated in a series of studies focusing on brain connectivity established via functional and diffusion tensor imaging (Ameis et al., 2011; Cheng, Rolls, Gu, Zhang, & Feng, 2015; Courchesne & Pierce, 2005; Di Martino et al., 2011; Just, Cherkassky, Keller, Kana, & Minshew, 2007; Solso et al., 2016; Sundaram et al., 2008; Supekar et al., 2013; Uddin, Supekar, & Menon, 2013; Yao et al., 2016). For example, children and young adolescents with ASD showed abnormalities in both functional and structural connectivity, manifested as, for example, over‐connectivity in local circuits (Courchesne & Pierce, 2005; Keown et al., 2013; Solso et al., 2016), and underconnectivity between distant brain regions (Abrams et al., 2013; Barttfeld et al., 2011; Just et al., 2007; Sundaram et al., 2008). Persistent alterations in functional and WM connectivity were also observed in young adults with ASD (Arnold Anteraper et al., 2018; Joshi et al., 2017; Mengotti & Brambilla, 2014; Tyszka, Kennedy, Paul, & Adolphs, 2014), though these changes may generally reduce with the maturation in individuals with ASD (Uddin et al., 2013). In addition, by utilizing graph‐theoretic approaches, randomized network organization (Barttfeld et al., 2011; Itahashi et al., 2014; Rudie et al., 2013) and altered hub topology (Itahashi et al., 2014; Ray et al., 2014) were found in children and adults with ASD. These findings suggested ASD was associated with abnormal connectivity and network changes that can be used as markers.

Compare with functional and tractography‐based networks, the network of anatomical covariance, constructed by measuring the correlations of morphological features (volume, cortical thickness [CT], SA, etc.) between pairs of brain regions (Evans, 2013), is able to characterize the topological organization in a morphological perspective. For example, the anatomical network‐based “small‐world” organization measures the network organization relative to a matched randomly wiring network (He, Chen, & Evans, 2007; He, Chen, & Evans, 2008; Zheng, Yao, Xie, Fan, & Hu, 2018), and modular architecture measures the organization pattern of the sparsely interconnected communities in the network (Chen, He, Rosa‐Neto, Germann, & Evans, 2008). The positive anatomical correlations were partially (35–40%) mediated by fiber connection (Gong, He, Chen, & Evans, 2012), suggesting that the anatomical correlations contained unique information and may partially reflect the connecting WM pathways. In fact, previous studies have found that ASD affected the cortical GM in terms of the modular organization in autistic children (Shi, Wang, Peng, Wee, & Shen, 2013) and intrinsic connectivity in adults with ASD (Ecker, Ronan, et al., 2013). Moreover, the cortico‐cortical GM connectivity between brain regions can be used to identify individuals with ASD from typically developed persons (Wee, Wang, Shi, Yap, & Shen, 2014; Zheng, Eilamstock, et al., 2018). Therefore, the network features may provide unique information for ASD that may not be characterized by individual morphological features alone. Though changes in GM connectivity within a specific age range (e.g., children) have been extensively studied, the alteration of large‐scale GM network with the development of autistic brains remains unclear.

In the present study, we aimed to investigate the developmental patterns of cortical GM topology in individuals with ASD from childhood to adulthood. T1‐weighted (T1w) images of magnetic resonance imaging (MRI) of 17 acquisition centers from the Autism Brain Imaging Data Exchange (ABIDE) database (http://fcon_1000.projects.nitrc.org/indi/abide/) were used to construct networks based on both CT and SA for individuals with ASD (246 subjects) and the typically developing controls (TDCs) (246 subjects). We characterized the topological differences between them in three age ranges, including childhood (7–11 years), adolescence (12–17 years), and adulthood (18–29 years). Graph‐theoretic measures were calculated, including small‐worldness, hub nodes, and modular structure, to provide a comprehensive picture of the ASD‐related alterations in structural network organization at different developmental stages. In addition, we brought up a center‐paired permutation strategy that avoided possible bias occurred during centers resampling to assess the group differences in network properties.

## 2 MATERIALS AND METHODS

### 2.1 Images

T1w images were obtained from the ABIDE I database (http://fcon_1000.projects.nitrc.org/indi/abide/), which were acquired on 3 T scanners of 17 international imaging centers (13 from the United States and 4 from Europe) at a resolution of 1 × 1 × 1 mm^3^. Detailed acquisition parameters are available at the ABIDE website. Image acquisition at each site was approved by its local institutional review board. Participants in the ASD group were diagnosed by experienced clinicians using Autism Diagnostic Interview‐Revised (ADI‐R), the Autism Diagnostic Observation Schedule, and/or DSM‐IV‐TR. The typical developing individuals had no reported personal or family history of ASD and were matched at the group level to ASD relative to age. Participants were excluded if they had psychiatric or neurological disorders. Details of diagnostic criteria at each center were shown in Table S1. All images were checked before preprocessing to ensure that all participants were free of brain injury.

We included individuals with ASD who were left‐handed, younger than 30 years old at the scan, and in the high‐functioning end of the spectrum (full‐scale IQ ≥80) for analysis. Subjects with low‐quality MRI scans were excluded (see Section 2.2). The qualified ASD subjects were categorized into three cohorts based on the age at scan: children (7–11 years), adolescents (12–17 years), and adults (18–29 years) (Aboud et al., 2019; Knoppert et al., 2007). For each individual with ASD, we selected a TDC subject from the same acquisition site with matched age, gender, handedness, and full‐scale IQ. Specifically, we first selected the TDC subjects with the same gender and handedness as the target individual with ASD; then we picked several TDC subjects whose age were very close to the target subject; we chose the final TDC subject with comparable FIQ (relative to the target ASD subject) from these age‐matched TDCs. The absolute mean between‐group differences of age and FIQ of the selected child/adolescent/adult subjects were 0.30/0.32/0.62 (*SD* = 0.33/0.37/0.74) and 14.86/13.39/12.92 (*SD* = 11.27/10.55/11.11), respectively. The ASD or TDC individuals who had no matched subjects in the other group were excluded. Finally, a total of 246 subjects with ASD, including 75 children, 91 adolescents, 80 adults, and 246 matched neurotypical controls were selected. The distribution of the diagnostic categories, including autism, Asperger's disorder, and pervasive developmental disorder not‐otherwise‐specified, of the selected ASD individuals is illustrated in Figure S1. Basic information regarding the demographics of the participants and the acquisition sites are given in Tables 1 and 2, respectively. No significant difference was found in age and full‐scale IQ between ASD and matched TDC groups for each age range (*p*s > .05).

**TABLE 1:** Demographic information of participants

	Age		Gender		Full‐scale IQ	
Group	Mean	*SD*	Male	Female	Mean	*SD*
Children (7–11 years)						
ASD	9.81	1.25	66	9	107.85	15.91
TDC	9.88	1.30	66	9	113.53	11.25
Adolescents (12–17 years)						
ASD	14.50	1.56	79	12	106.40	14.15
TDC	14.53	1.49	79	12	108.19	11.83
Adults (18–29 years)						
ASD	22.84	3.36	71	9	110.04	13.62
TDC	22.90	3.31	71	9	114.63	11.42

Abbreviations: ASD, autism spectrum disorder; *SD*, standard deviation; TDC, typically developing control.

**TABLE 2:** Information of acquisition centers of the selected data

Acquisition center	Children		Adolescents		Adults	
	ASD	TDC	ASD	TDC	ASD	TDC
CALTECH					7	7
CMU					7	7
KKI	12	12				
LEUVEN1					9	9
MAX_MUM	4	4			4	4
NYU	22	22	11	11	12	12
OHSU	6	6				
OLIN			6	6	4	4
PITT			10	10	7	7
SBL					2	2
SDSU			8	8		
STANFORD	8	8				
TRINITY			13	13	8	8
UCLA1	7	7	17	17		
UCLA2	2	2	3	3		
UM1	8	8	8	8		
UM2			8	8		
USM					20	20
Yale	6	6	7	7		

Abbreviations: ASD, autism spectrum disorder; CALTECH, California Institute of Technology; CMU, Carnegie Mellon University; KKI, Kennedy Krieger Institute; LEUVEN, University of Leuven; MAX_MUM, Ludwig Maximilians University Munich; NYU, New York University Langone Medical Center; OHSU, Oregon Health and Science University; OLIN, Olin Center, Institute of Living at Hartford Hospital; PITT, University of Pittsburgh; SBL, Social Brain Lab, BCN Neuroimaging Center, University Medical Center Groningen; SDSU, San Diego State University; STANFORD, Stanford University; TRINITY, Trinity Center for Health Sciences; UCLA, University of California, Los Angeles; UM, University of Michigan; USM, University of Utah; Yale, Yale Child Study Center.

### 2.2 Image preprocessing

All image data were preprocessed using FreeSurfer v5.3.0 (http://surfer.nmr.mgh.harvard.edu). Briefly, the preprocessing included motion correction, exclusion of nonbrain tissue (Ségonne et al., 2004), coordinate transformation, intensity normalization, segmentation, and generation of GM–white matter boundary (Dale, Fischl, & Sereno, 1999; Fischl, Sereno, & Dale, 1999). Low‐quality MRI scans that failed the segmentation or showed segmentation inaccuracies between the generated GM–white matter boundary were excluded (26 ASD subjects and 3 TDCs in total). Surfaces were inflated and registered to a priori template to calculate the morphological measurements of the brain cortex. Here, two commonly used morphological measures, including SA and CT, were extracted. The reasons for choosing SA and CT were because these two measures were key features for characterizing cortical thinning and surface shrinking that have been widely reported in individuals with ASD (Ecker, Ginestet, et al., 2013; Hyde, Samson, Evans, & Mottron, 2010; Libero, DeRamus, Deshpande, & Kana, 2014; Mak‐Fan, Taylor, Roberts, & Lerch, 2012; Wallace, Dankner, Kenworthy, Giedd, & Martin, 2010), and they represented different information of cortical morphology compared to other features (e.g., vertical volume) (Panizzon et al., 2009; Sanabria‐Diaz et al., 2010). Thus, these two measures were chosen to reveal the ASD‐related changes in morphological topology from distinct domains. The SA at each vertex was the average of all triangular faces surrounding the vertex (Winkler et al., 2012); and the CT was measured as the closest distance between the WM and GM surface at each vertex (Fischl & Dale, 2000). Each cortical surface was parcellated into 148 regions according to the Destrieux atlas (2009) (Destrieux, Fischl, Dale, & Halgren, 2010) in FreeSurfer. Regional SA, which represented as the 2D flattened surface of the brain region, and regional average CT were then computed.

### 2.3 Construction of networks of anatomical covariance

**FIGURE 1:**
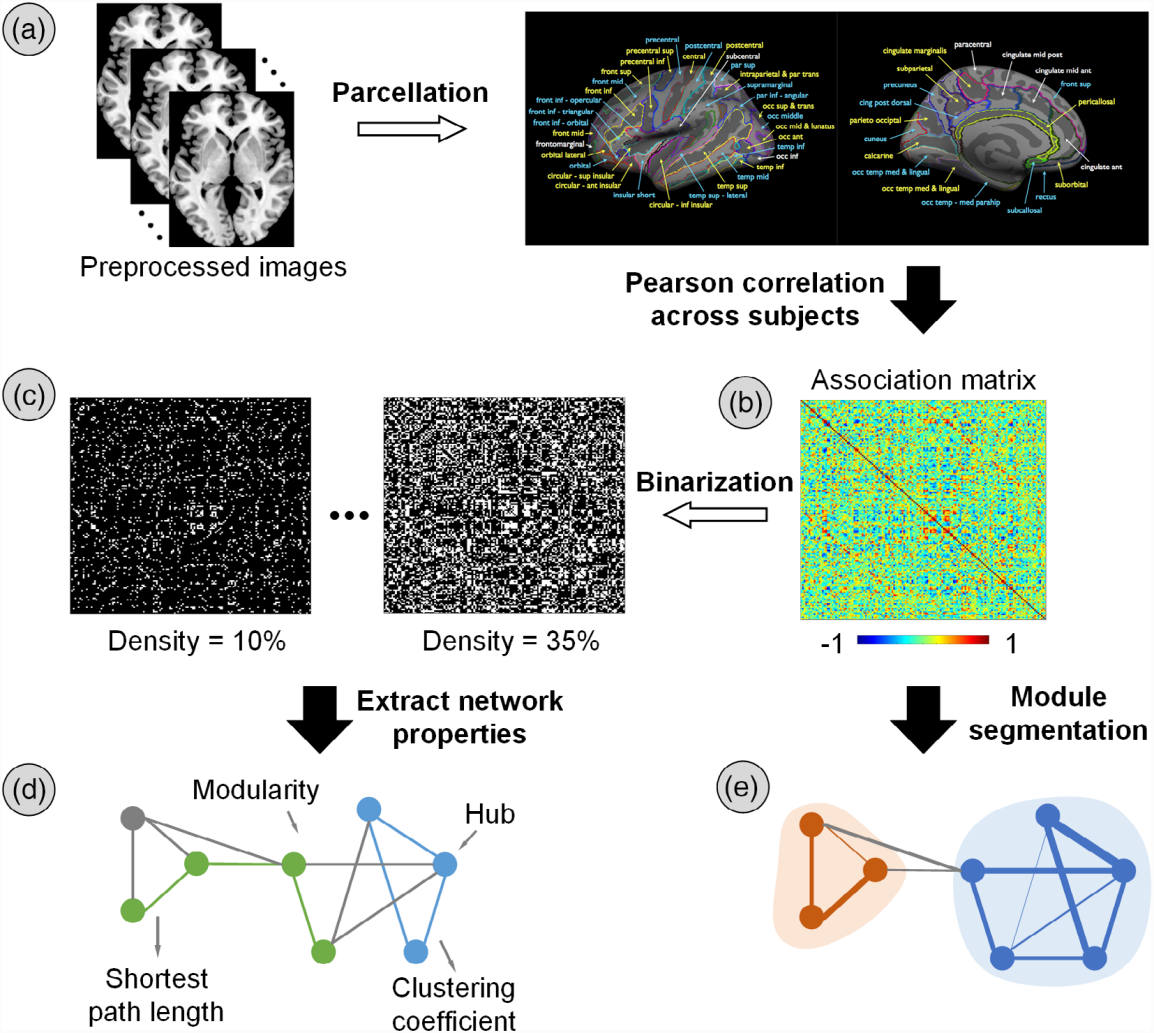
The pipeline of network analysis. All preprocessed T1‐weighted images were registered to a prior template including 148 brain regions, and the regional average morphological measures (i.e., cortical thickness [CT] and surface area [SA]) were extracted, yielding an N‐participants × 148 data matrix. Before network construction, regional data of each acquisition center were rescaled, and the effects of age, gender, and global CT/SA were regressed out from the rescaled regional measures. The structural connectivity between pairs of brain regions was estimated by calculating Pearson correlations across individuals, separately for autism spectrum disorder (ASD) and TDC cohorts, and separately for different age bands. The optimal module partitions were determined based on the weighted correlation matrix after removing all negative connections. The correlation matrix was then binarized by retaining the strongest 8–35% of connections in 1% increments. Network properties were calculated at each link density

To reduce the differences in feature scaling between different sites, we rescaled the surface measures of each selected subject using the median absolute deviation (MAD) of all preprocessed data from the same acquisition site after quality control (but before the selection of age, sex, and FIQ matched individuals) (Wulff & Mitchell, 2018; Zheng, Woo, et al., 2019). For each site, we calculated the MAD of regional properties (CT or SA) of each brain region across participants, and the regional properties of each participant were then rescaled by dividing the MAD of this region, resulting in a ratio of MAD. Linear regression was performed on the regional measures to control for the effects of age, gender, and global average SA/CT in each age group (i.e., child, adolescent, and adult), separately (He et al., 2007; Yao et al., 2010; Yao et al., 2015). The residuals of the linear regressions were used to construct the cortical structural network. Here, each brain region represented a node of the network, and the edge between every pair of nodes was defined as the Pearson correlation coefficient between the structural measures (i.e., SA and CT) of the two regions across subjects (He et al., 2007; He et al., 2008). For each group, a 148 × 148 symmetric association matrix (10,878 total connections) was obtained. The pipeline for network construction and analysis is shown in Figure 1.

### 2.4 Network analysis

#### 2.4.1 Network metrics

Network metrics were calculated based on the binary association matrix, where the edges were set to 1 if they exceeded a predetermined threshold and 0 if they below the threshold. To characterize the robustness of our analyses as a function of link density, we performed analyses by varying the network sparsity, from 8 to 35% in 1% increments by increasing the threshold. This range was chosen because the networks were fully connected at 8% sparsity and became randomly organized when the density was above 35% (see Figure S2). All self‐connections and negative connections were excluded from analyses. Network analyses were performed using the Brain Connectivity Toolbox (Rubinov & Sporns, 2010), and the results were visualized via BrainNet Viewer (Xia, Wang, & He, 2013).

#### 2.4.2 Global network properties

To compare the overall organization of networks between ASD and TDC for each age range, graph theoretical analysis was utilized to extract four common properties from the graph for both ASD and TDC cohorts at each link density, including clustering coefficient, global efficiency, small‐worldness, and modularity. The clustering coefficient of a node is defined as the number of suprathreshold edges between the node's neighbors divided by all possible edges between its neighbors. The characteristic path length (*L*) is the smallest number of connections between pairs of nodes, averaged across all pairs. Because a longer route, on average, from node to node leads to lower efficiency of information transfer, the measure of global efficiency of a graph is defined as the inverse of *L*. A small‐world network typically shows higher clustering and comparable *L* relative to a random graph (Watts & Strogatz, 1998). The modularity measures the extent to which a graph can be segmented into nonoverlapping communities with maximization of intra‐module edges and minimization of intermodule edges (Newman, 2006).

#### 2.4.3 Hub analysis

Eigenvector centrality was used to measure the importance of nodes in the network. The principle of eigenvector centrality is that links connecting to important nodes are worth more than connecting to others, which does not only take into account the connectedness of a node itself (i.e., its degree) but also sensitive to more complicated situations, for example, a high degree node connecting to a number of low degree nodes or a low degree node connecting to a number of high degree nodes (Bonacich, 2007). Thus, eigenvector centrality provides a more comprehensive assessment when the centrality of a network is driven by differences in degree (Bonacich, 2007; Solá et al., 2013). To obtain a unified hub topology for each group across multiple link densities, we averaged the eigenvector centrality of each node over different link densities (from the strongest 5 to 35% of links in 5% increments) and defined the hub regions as nodes with z‐scored average eigenvector centrality >1.5 (Cohen & D'Esposito, 2016; Lynall et al., 2010; Zheng, Woo, et al., 2019). To explore whether the structural changes of a brain region could affect the nodal function in the cortical network, we calculated the Pearson correlation between regional structures and eigenvector centralities of ASD and TDC cohorts at each age range.

#### 2.4.4 Network assortativity

We further calculated the assortativity coefficient to investigate whether ASD influenced the assortative mixing of the cortical network. The assortativity measures the relationship between the strength of linked nodes (eigenvector centrality) on each side of the connectivity (Newman, 2002). In other words, nodes in an assortative network are inclined to connect with other nodes with similar strength, for example, hub nodes are more strongly clustered with other hub nodes, making the network robust against disruption (Bassett et al., 2008; Newman, 2002). Mathematically, assortativity is defined as:

$$r=\frac{\frac{1}{M}\sum_{i}j_{i}k_{i}-{\left[\frac{1}{M}\sum_{i}\frac{1}{2}\left(j_{i}+k_{i}\right)\right]}^{2}}{\frac{1}{M}\sum_{i}\frac{1}{2}\left({j_{i}}^{2}+k_{i}^{2}\right)-{\left[\frac{1}{M}\sum_{i}\frac{1}{2}\left(j_{i}+k_{i}\right)\right]}^{2}}$$

where *j*_*i*_ and *k*_*i*_ are the eigenvector centralities of the nodes at the ends of the *i*th edge and *M* is the number of edges in the network. Here, the averaged eigenvector centralities over link densities were used as nodal strength for calculation.

#### 2.4.5 Community detection

To avoid the effect of network sparsity on modular partitions, we performed the community detection algorithm on the weighted network following the pipeline described in (Cohen & D'Esposito, 2016) (Figure 1). Briefly, the Louvain community detection algorithm was utilized to estimate the optimal partition of nodes in the network that had only positive weighted edges (Blondel, Guillaume, Lambiotte, & Lefebvre, 2008). Because the partitions may vary from run to run, the algorithm was repeated 150 times to yield a consensus matrix (*D*), where *D*_*ij*_ indicated the probability that node *i* and node *j* were assigned to the same community. The agreement value was set to zero if the probability that a pair of nodes were assigned to the same community was lower than 50%. We then ran the Louvain algorithm 100 times on the consensus matrix (*D*) to compute one consensus modular partition. This step was repeated until the single consensus partition was obtained. This method was shown to be more reliable than other commonly used algorithms (Lancichinetti & Fortunato, 2012).

#### 2.4.6 Modular segregation

We applied the segregation index (SI) to measure the degree of segregation of each module (Chan et al., 2018), defined as:

$$\mathrm{SI}=\frac{{\bar{Z}}_{w}-{\bar{Z}}_{b}}{{\bar{Z}}_{w}}$$

where ${\bar{Z}}_{w}$ is the average of Fisher's z‐transformed connections within a specific module, and ${\bar{Z}}_{b}$ is the average z value of connections between this module and all the other modules. To compare the SI of each module of ASD cohort with the TDCs, we extracted the SI of TDC cohort by applying the modular partition of ASD to the TDCs, with the null hypothesis that the segregation of this module in ASD cohort was at the same level as in the TDCs.

### 2.5 Statistical analysis

The statistical differences of network properties between individuals with ASD and the TDCs within each age range were tested using nonparametric permutation tests with 5,000 permutations. In each permutation, we randomly reallocated subjects to ASD or TDC group and built the association matrices for the randomized groups (He et al., 2008; Yao et al., 2010). Because the dataset of each age range included samples from over nine acquisition centers, to reduce the site bias that may occur during permutation (e.g., all samples from one acquisition center may have the chance to be reassigned to one group), we performed a center‐paired permutation test by permuting samples within each of the acquisition centers and the outcomes of all the centers were put together to form a final pair of randomized groups. Then, the network properties of the randomized groups were computed, and the between‐group differences of these properties were calculated. This procedure was repeated 5,000 times to generate the confidence interval (CI) for each network property at every density, and two‐tailed, uncorrected *p* values were calculated from the CI. This paired permutation strategy was compared with the regular permutation regardless of the site difference. To investigate whether the center bias was well controlled, one‐sample *t* test was used to compare the corrected data of each acquisition center with the mean value of all the centers. This step was performed for each group within different age ranges. Multiple comparisons and correlations were corrected by false discovery rate (FDR) corrections at the level of *q* = 0.05.

## 3 RESULTS

### 3.1 Differences in the overall network topology between ASD and the TDC groups

We first tested whether the SA or CT in individual parcels (e.g., average SA/CT of the parcels) or vertex were different between ASD and TDC groups, and did not find significant between‐group differences in any of the three age ranges (*q*s > 0.05, FDR corrected), suggesting the group differences were relatively mild and could not be detected with parcel‐based analysis.

**FIGURE 2:**
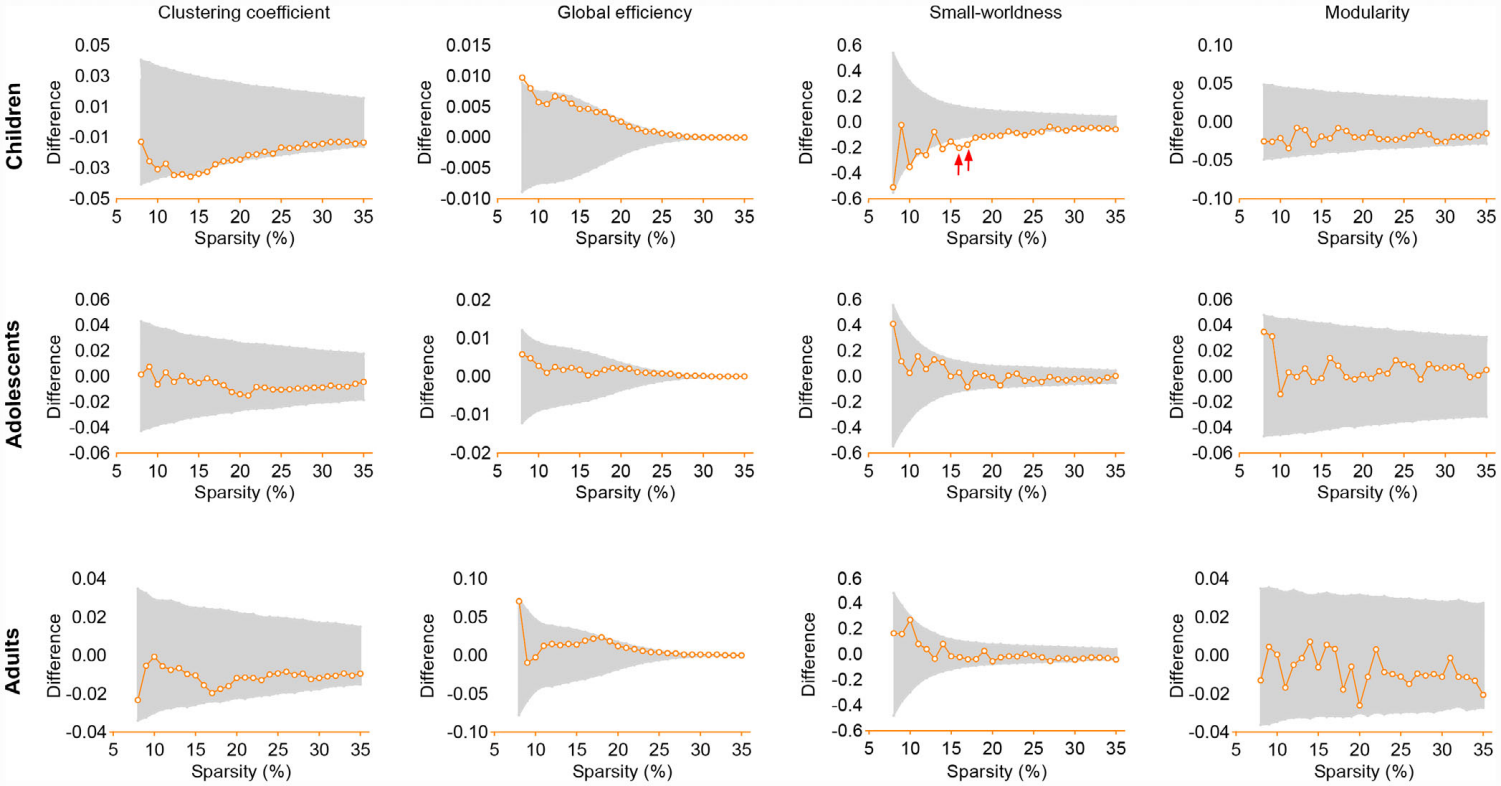
Comparison of properties of surface area (SA) network between individuals with autism spectrum disorder (ASD) and the TDCs with varying connective sparsities, in different age bands. The gray shade shows the 95% confidence interval obtained from 5,000 permutations, and the group differences were presented in orange at varying network sparsities. Small‐worldness of children with ASD significantly decreased at link sparsities of 16 and 17% (red arrows, *q* < 0.05, false discovery rate [FDR] corrected)

We then checked the global network properties. Networks of both SA and CT covariation were “small‐world,” and the small‐worldness (*σ*) of the CT network was slightly higher than the SA network in all three age groups (Figure S2). Results from the permutation test showed that the small‐worldness of the SA network was significantly reduced in children with ASD at 16–17% link density when compared to the matched TDCs (*p* < .05, FDR corrected, Figure 2). However, the small‐worldness did not show a significant change in either adolescents or adults with ASD. Clustering coefficient, global efficiency, and modularity of the SA network in the ASD cohort did not significantly differ from those in the neurotypical cohort in any of the age groups. In addition, the properties of the CT network did not exhibit evident alteration in individuals with ASD (Figure S3), suggesting that ASD may have less effect on the topologic organization of CT than that on SA. Therefore, further analyzes only focused on the network of SA covariation.

We also applied linear regression to control site information (Haar, Berman, Behrmann, & Dinstein, 2014) and compared the results with the aforementioned analysis using MAD rescaling. Both approaches performed well in controlling site bias of SA (one‐sample *t* test, *q*s > 0.05, FDR corrected), and MAD rescaling achieved lower variance at all acquisition centers (Figure S4), suggesting the results derived from SA were not significantly biased among the sites. However, the site bias of CT cannot be completely corrected by either approach, as we found the corrected CT in 7 out of 17 sites were significantly differed from the average of all the sites in different age ranges (one‐sample *t* test, *q*s < 0.05, FDR corrected). This may due to the large variance between the original CT distributions across acquisition sites and was consistent with a recent study showing CT was less reliable than SA and volume (Carmon et al., 2020). Though this may not affect the main results of this study, the site difference remained a concerning factor as in many other multicenter studies (Grech‐Sollars et al., 2015; Haar et al., 2014; Teipel et al., 2017; Zheng, Eilamstock, et al., 2018), and should be carefully examined. In addition, we compared the center‐paired permutation strategy and the regular permutation strategy (permute all samples regardless of centers). Figures S5 and S6 showed the paired permutation strategy performed better in controlling possible center bias during the permutation process and did not increase the false‐positive rate.

### 3.2 Changes of hub topology in ASD and TDC cohorts

**FIGURE 3:**
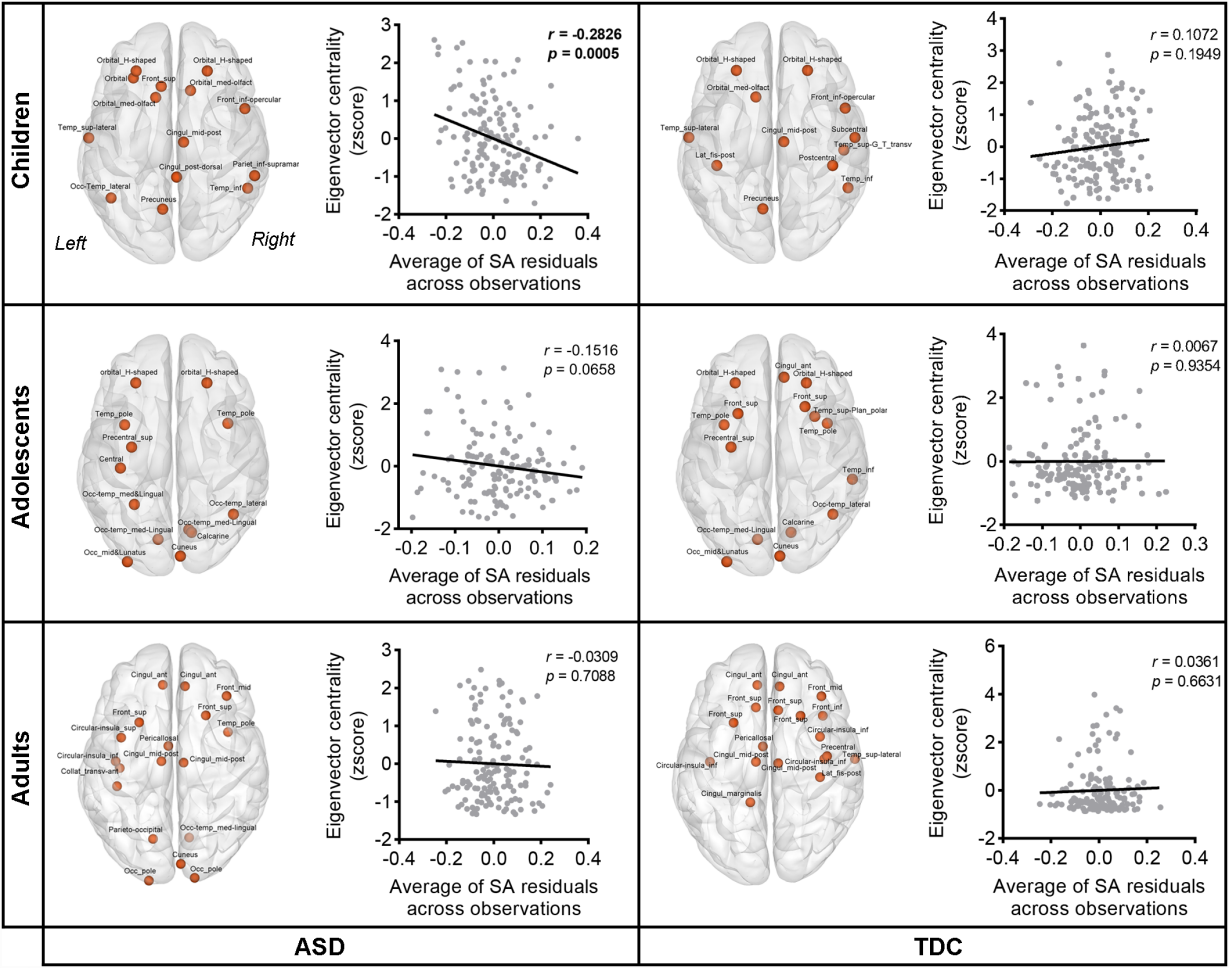
Spatial distribution of hub regions and the association between the population‐averaged regional surface areas (SAs) and eigenvector centralities in autism spectrum disorder (ASD) and TDC groups in three age bands. Hub regions were defined as z‐scored eigenvector centrality >1.5. For correlation analysis, the effects of age and gender were regressed out from the regional SA, and the residuals of each brain region were then averaged across participants to correlate with the z‐scored eigenvector centrality of each region. Pearson correlations were calculated for each age basket, respectively. The SA showed significant negative correlation with eigenvector centrality across regions in children with ASD (*r* = −.2826, *q* < 0.05, false discovery rate [FDR] corrected)

The hub regions (z‐scored eigenvector centrality >1.5) in the ASD and TDC cohorts at each age basket are visualized in Figure 3a. No significant between‐group differences in eigenvector centrality were found (*q*s > 0.05, FDR corrected). To provide a clearer visualization of the alteration pattern in nodal centrality of the ASD cohort, we plotted the ratio changes of eigenvector centrality of all nodes relative to the TDCs (Figure S7). Similar hub topology was found in both children with ASD and the neurotypical children, except the orbitofrontal cortex where more hubs were identified in the ASD cohort (e.g., bilateral H‐shaped orbital sulcus and olfactory cortex). For the adolescent group, nodes with relative lower centrality were mainly placed in the parietal cortex, and most of the hubs were located within frontal and occipital cortices for both the ASD and TDC subjects. For the adult group, hub regions of both ASD and TDC cohorts were localized in the anterior and middle brain, including the prefrontal cortex (PFC), anterior‐to‐middle cingulate gyri, and insula‐opercular cortex; furthermore, some nodes within the left operculum and parieto‐occipital cortex (e.g., cuneus and occipital pole) were identified as hubs in ASD group.

**FIGURE 4:**
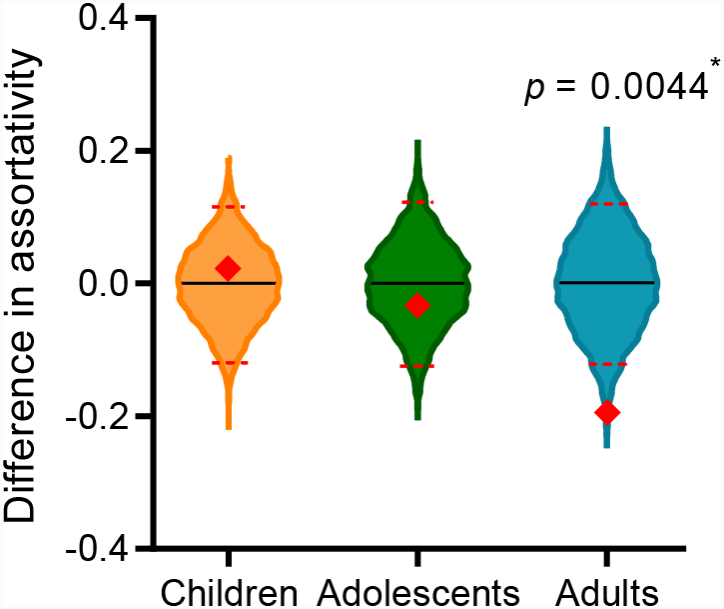
The difference in network assortativity between individuals with autism spectrum disorder (ASD) and the TDCs. The assortativity of adults with ASD significantly decreased relative to the TDCs (*q* < 0.05, permutation test, false discovery rate [FDR] corrected). Blackline and dotted red lines are the mean and 95% confidence interval of the group difference, respectively, obtained from 5,000 permutations. Red diamonds indicate the observed statistic

Interestingly, the eigenvector centrality significantly negatively correlated with regional SA in children with ASD (*r* = −.2826, *q* < 0.05, FDR corrected, Figure 3), suggesting that the alteration of SA of a brain region may influence the role of that region played in the whole network. However, the correlations were nonsignificant in either adolescents and adults with ASD or the three TDC groups (*q*s > 0.05, FDR corrected). We also found that there was a significant reduction in network assortativity in adults with ASD compared to the matched controls (*q* < 0.05, permutation test, FDR corrected, Figure 4), suggesting hub nodes tended to cluster more with nonhub nodes and cluster less with other hubs in adults with ASD.

### 3.3 Different modular organizations of the cortical SA network in ASD and TDC cohorts

**FIGURE 5:**
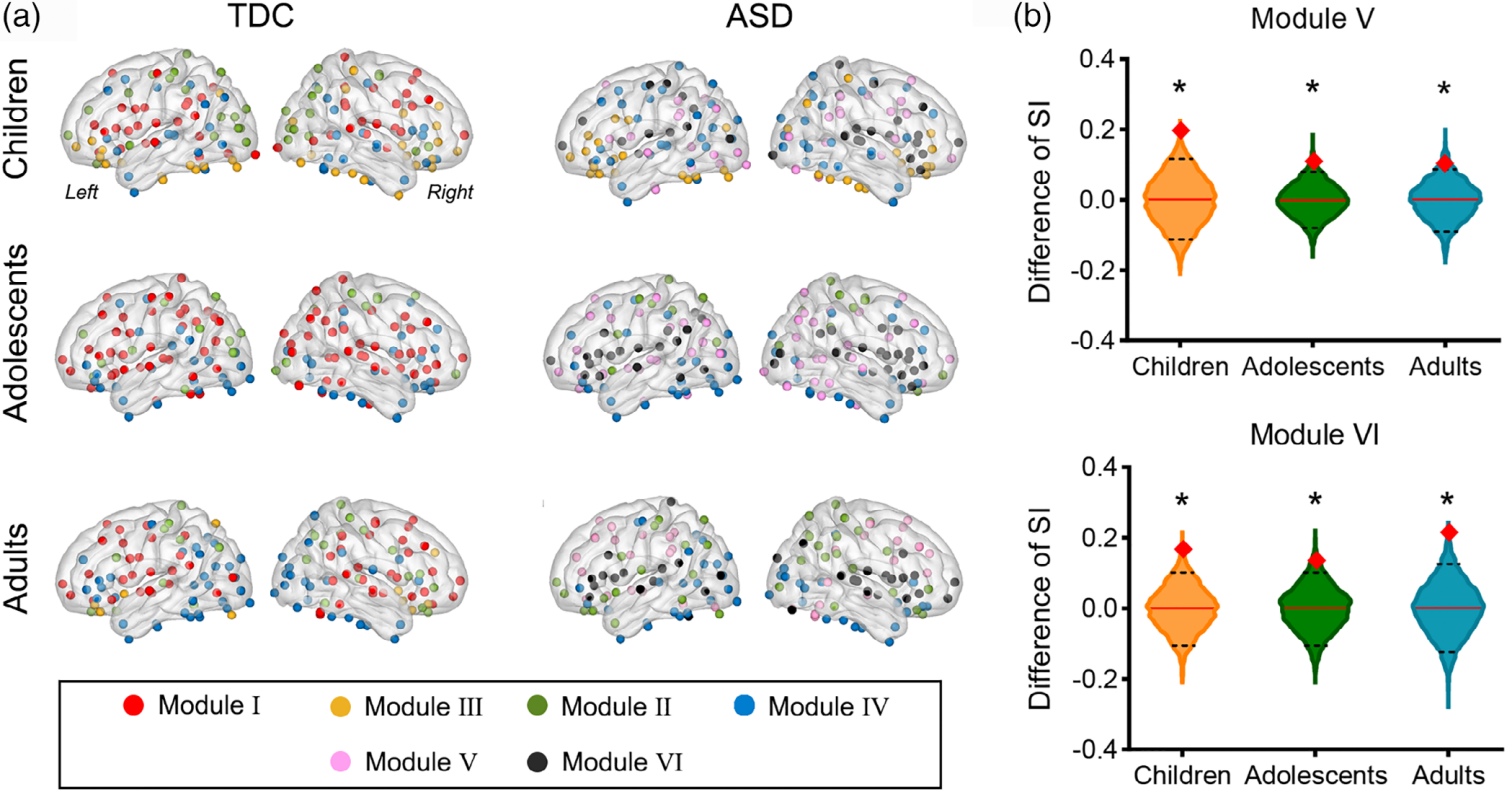
Modular organization of the network of surface area (SA) covariance. (a) Visualization of modules of autism spectrum disorder (ASD) and TDC groups in the three age ranges. Colors indicate different modules. (b) The between‐group difference in the segregation index (SI) of Modules V and VI in three age groups. Increased SI of Module V and Module VI in individuals with ASD of all three age groups were found, relative to the matched TDCs (*q*s < 0.05, permutation test, false discovery rate [FDR] corrected). Redline and dotted black lines are the mean and 95% confidence interval of SI difference, respectively, obtained from 5,000 permutations. Red diamonds indicate the observed statistic

In the TDC groups, we identified four modules in children and adults, and three modules in adolescents (Figure 5a). Module I (in red) included PFC, insular‐opercular cortex, ACC and posterior cingulate cortex, angular gyri (ANG), precuneus, and parts of superior temporal gyri (STG), which was in good accordance with the distribution of the (DMN) and the “cognitive control network” (Allen et al., 2014; Park, Kim, & Park, 2014; Yeo et al., 2011) and showed high consistency in the three age ranges. Module II (in green) mainly included postcentral gyri, ventral frontal cortex, and parts of the occipital cortex. Module III (in yellow), which consisted of the ventral and orbital frontal cortex and parts of the inferior temporal cortex, were clearly observed in child and adult TDCs. Module III in the adolescents was merged with Module IV (in blue), which mostly located in the temporal and occipital cortices.

In the ASD group, two additional modules (Modules V and VI) that were not observed in the TDCs were identified in all the three age groups (Figure 5a). Specifically, Module V (in pink) mainly included PFC, precuneus, ANG, and several superior and inferior temporal regions; and Module VI (in black) covered the anterior‐to‐posterior cingulate gyri, insular‐opercular cortex, and posterior STG. The SI of Modules V and VI consistently increased in individuals with ASD in all three age ranges (*q*s < 0.05, permutation test, FDR corrected, Figure 5b). These results indicated an evident reorganization of modular structure in the SA covariation network of individuals with ASD, despite the nonsignificant between‐group differences in modularity magnitude.

## 4 DISCUSSION

The structural development of the autistic brain across the human lifespan has long been an open question. Although studies have reported significant changes of cortical morphology in individuals with ASD, for example, brain overgrowth in early childhood (Courchesne et al., 2003; Courchesne et al., 2007; Heather Cody Hazlett et al., 2017; Zwaigenbaum et al., 2014) and accelerated cortical thinning in adulthood (Braden & Riecken, 2019; van Rooij et al., 2018), and variations in cortical SA (Ecker et al., 2014; Ecker, Ginestet, et al., 2013; Hazlett et al., 2011; Libero et al., 2014; Mak‐Fan et al., 2012) and GM volume (Eilam‐Stock et al., 2016; Hyde et al., 2010; Libero et al., 2014; Riddle, Cascio, & Woodward, 2017), these findings remain largely inconsistent (Ecker, Bookheimer, & Murphy, 2015; Haar et al., 2014; Nickl‐Jockschat et al., 2012). By utilizing machine learning technology, studies have reported limited utility of morphological measures for ASD diagnosis on a large sample set (Haar et al., 2014). On the other hand, atypical cortico‐cortical GM connectivity that was reported in both children and adults with ASD (Ecker, Ronan, et al., 2013; Shi et al., 2013) can significantly enhance the diagnostic performance (Wee et al., 2014; Zheng, Eilamstock, et al., 2018), which would also help to understand the developmental pattern of the autistic brain.

The current study extended previous works by showing ASD‐related changes in cortico‐cortical structural covariation in childhood, adolescence, and adulthood. Our results suggested that ASD (a) resulted in substantial changes in cortical structural organization in the childhood, including reduced small‐worldness, centralized hubs in the orbitofrontal cortex, and reduced network assortativity in the adulthood; and (b) caused disordered module organization that formed a specific module including brain regions associated with socio‐emotional processing and cognitive functions (e.g., insular‐opercular cortex, ACC, and STG). These changes implied that although the individual morphological features (e.g., SA and CT) may have less abnormal changes in individuals with ASD, the organization of cortical topology remained largely abnormal, which may explain the structural basis for the impaired behavioral and social–emotional functions in ASD cohort.

Though connectivity changes are originated from anatomical alterations that may result from various latent processes (e.g., maturation, aging, and disease), they reflect unique information that may not be captured by using anatomical features alone, because they better characterize the distributed influence of disease in the brain (Evans, 2013). The true advantage of structural network analysis lies in the quantitative descriptions of structural changes in both cortico‐cortical associations and high‐level topological structures, which may complement the understanding of the ASD mechanism from an anatomical perspective. Furthermore, the effects of ASD on the cortical topological organization during childhood, adolescence, and adulthood are largely uncharted territory, which is important for the etiology of ASD. Thus, the main contribution of this study was to demonstrate the alteration pattern of cortical topological in individuals with ASD at different age ranges, which revealed a more comprehensive description than using the morphological features alone.

### 4.1 Network of SA covariations revealed the alteration of cortical organization in autistic children rather than surface morphology

The pathological process that underpins the early enlargement of the cortex remains unclear. Interestingly, we showed ASD significantly influenced the organization of the network of SA, but not CT, covariation in childhood. This was consistent with the findings that early enlargement of brain volume in ASD was driven by an expansion of SA (but not CT) (Hazlett et al., 2011). Actually, the covariation matrix of CT and SA may reflect distinct aspects of the interaction between brain structures (Sanabria‐Diaz et al., 2010) that may be caused by multiple factors (e.g., genetic (Panizzon et al., 2009) and microstructure (Pontious, Kowalczyk, Englund, & Hevner, 2008)). Our results suggested the network of SA covariance in children with ASD showed a more randomized organization than that in typically developing children. Because small‐world topology reflects an optimal balance between global integration and local segregation (Liao, Vasilakos, & He, 2017; Sporns & Zwi, 2004), the reduction of small‐worldness may indicate a disturbance of this balance. Similar result has been reported in functional MRI study in a similar age range (Rudie et al., 2013). However, the small‐worldness tended to be normalized in adolescents and adults with ASD. This may result from the arrested growth or decrease in brain volume in older age (Courchesne et al., 2011). Although the atypical growth was reported mostly at the age before 4 years and disappear at age of 6–8 years (Courchesne et al., 2001), we speculated that the reduced morphological changes may still have long‐lasting influences on topological structure, as we found reduced small‐worldness in children with ASD at the older age (age of 7–11 years), although their CT and SA did not significantly differ from healthy controls.

### 4.2 Altered hub topology of adults with ASD suggested the persistent effects of ASD on adult brain morphology

Compared to the neurotypical adults, regions within the occipital cortex played more important roles in adults with ASD, who also demonstrated decreased network assortativity, though the regional CT and SA measurements in adult ASD subjects returned to normal levels. These results were partially consistent with a previous functional MRI study showing that compared to TDCs, adults with ASD were accompanied by decreased centrality in right PFC and increased centrality in parieto‐occipital and posterior occipital cortices (Itahashi et al., 2014). In contrast, hubs of the SA network of the TDCs showed localized distribution in adulthood, mainly within medial prefrontal and lateral frontoparietal cortices (e.g., frontal and cingulate cortices, and pars opercularis). This pattern differed from the hub distribution reported in networks of CT covariance (Bernhardt, Chen, He, Evans, & Bernasconi, 2011; He et al., 2007), but was in line with previous volumetric network studies showing increased hubs in anterior, medial, and lateral prefrontal cortices in young and middle‐aged adults (Palaniyappan et al., 2019; Palaniyappan, Park, Balain, Dangi, & Liddle, 2015) and localized hub topology in the adulthood (Li et al., 2013). Possible interpretations for this phenomenon were that SA explained most of the changes in cortical volume in adults with ASD (Ecker, Ginestet, et al., 2013) and showed different attributes in structural brain networks relative to CT (Sanabria‐Diaz et al., 2010). The diffusive distribution of hub nodes (relative to the matched TDCs) and reduced network assortativity implied that the adult brains may experience broad influence from ASD and the hub regions were more inclined to connect with nonhub regions, making the network vulnerable to disruptions in adulthood (Bassett et al., 2008; Newman, 2002).

Interestingly, we found that regional SA was able to predict the importance of brain regions in the network in children with ASD, with larger SA accompanied with lower eigenvector centrality; however, this relationship was not statistically significant in other age groups of ASD, nor the TDCs. This phenomenon, we speculated, may be associated with the overdevelopment of the autistic brain in early childhood that altered the hub topology, and children with ASD (aged 7–11) may still experiencing the sequelae of the atypical development, making the less affected areas took more important position in the cortical network (e.g., the orbital cortex has been indicated with less changes in children with ASD (Carper & Courchesne, 2005)).

### 4.3 Modular reorganization impeded the integration of cognitive functions in the autistic brain

Another interesting finding was that individuals with ASD showed distinct modular partitions compared with the TDCs. In general, the alteration of modular structure in the anatomical network may result from common latent processes, such as growth. However, the reorganization occurred in the ASD group in all the three age bands, but not in the neurotypical controls who had matched age, sex, handedness, and IQ, providing strong evidence for ASD‐induced changes in network organization. Regions that belonged to the same module in the controls (Module I) reorganized into two segregated modules (i.e., Modules V and VI) in individuals with ASD, with significant SI increases in both of the two modules at different ages, suggesting increased coherence in SA changes within the reorganized modules but decreased coherence with regions outside the modules. These changes may result from the distinct development trajectory in the autistic brain. In addition, the detected modules were highly consistent with a previous study showing segregation between the PFC and insula/STG in children with ASD (Shi et al., 2013), but differed from the finding of another study (Bethlehem, Romero‐Garcia, Mak, Bullmore, & Baron‐Cohen, 2017) which showed high modular overlapping between autistic and neurotypical children. The inconsistency may due to the differences in the features under examination, sample size, and module detection algorithms (Carmon et al., 2020; Taya, de Souza, Thakor, & Bezerianos, 2016).

More broadly, the modular reorganization may be associated with the deficits of individuals with ASD in multiple cognitive functions (e.g., sensory processing, emotional and cognitive functions, and social cognition) (Allen & Courchesne, 2003; Baron‐Cohen, 2000; Eyler, Pierce, & Courchesne, 2012; Fan, 2012; Mackie & Fan, 2017; Perry, Minassian, Lopez, Maron, & Lincoln, 2007). The brain regions within Module VI (e.g., insula‐opercular cortex and STG) were primarily associated with emotional processing (Evrard, 2019; Gu, Hof, Friston, & Fan, 2013; Suzuki, 2012; Uddin, Nomi, Hébert‐Seropian, Ghaziri, & Boucher, 2017), language (Hickok & Poeppel, 2000, 2004; Scott, Blank, Rosen, & Wise, 2000; Scott & Wise, 2004), and social cognition (Fan, Chen, Chen, Decety, & Cheng, 2013; Lamm & Singer, 2010; Odriozola et al., 2015; Spagna et al., 2018; Uddin et al., 2017; Yamasaki et al., 2010; Zilbovicius et al., 2006). The isolation of this module (from Module I) implied disrupted integration in network architecture (e.g., impaired integration between attention, cognitive control, and socio‐emotional networks) (Müller, 2007) and reduced efficiency in information processing in autistic brains (Mackie & Fan, 2016; Rudie et al., 2013), which might be one of the factors contributing to the symptomatology of autism in social behavior (Kasari, Locke, Gulsrud, & Rotheram‐Fuller, 2011; White, Keonig, & Scahill, 2007), emotional processing (Ameis et al., 2011; Wicker et al., 2008), and language and communication (Tager‐Flusberg, 2003; Tager‐Flusberg, Paul, & Lord, 2005).

### 4.4 Limitations

There were several limitations in the current study. First, the data we used were acquired from multiple acquisition centers. This was a strength in the sense that it promoted the generalizability of our findings across observations. However, it may also bring unknown effects in the analysis, though we have strictly controlled the influence of this issue (e.g., the age, sex, handedness, and IQ were strictly matched between ASD and TDC groups, and data rescaling and center‐paired permutation strategy were adopted to mitigate the between‐site difference), the variabilities of diagnostic strategies and the experience of clinicians across centers may have potential influences and require attention in multicenter studies. In addition, the inconsistency between acquisition centers in different age baskets (e.g., the CMU center only has adult samples) also limited us to directly compare the network changes across age. Therefore, the developmental changes of the network properties across age groups in the present study were only qualitative and speculative and needed to be examined in future work. Second, the subcortical regions were excluded from our analysis, because of the definition of CT and SA was not appropriate for subcortical structures. Since some subcortical regions (e.g., amygdala (Baron‐Cohen et al., 2000) and thalamus (Nair, Treiber, Shukla, Shih, & Müller, 2013)) also play crucial roles in autism research, comparison of SA networks that include these regions would be important, but it remains as a challenge. Third, our results reflected the network changes at the group level, but network properties may vary across individuals in ways that cannot be captured by the current study. Recent studies have made it possible to build GM networks on individual‐person level (Tijms, Series, Willshaw, & Lawrie, 2012; Wee et al., 2014; Zheng et al., 2015; Zheng, Yao, et al., 2018; Zheng, Yao, et al., 2019), and these approaches can be utilized to characterize the alterations in cortical topology for the individual with ASD.

## 5 CONCLUSION

In conclusion, we found that ASD altered the topological architecture of SA but not CT, and caused a modular reorganization of the structural network during brain development from childhood to adulthood. We also found a significant reduction of small‐worldness in children with ASD, and this abnormality disappeared in older ages. Furthermore, hub regions of adults with ASD became dispersedly distributed across the brain and tended to connect with nonhub regions compared to the matched TDCs. These changes may reduce the robustness of the network and impede the integration of multiple cognitive functions, leading to the dysfunction in the autistic brain across the lifespan.

## CONFLICT OF INTEREST

The authors declare no conflict of interest.

## AUTHOR CONTRIBUTIONS

**Weihao Zheng**: Processed imaging data and performed all analyses with the preprocessed data. **Weihao Zheng**, **Zhiyong Zhao**, **Jin Fan**, and **Dan Wu**: Drafted the manuscript. All authors contributed to the interpretation and reviewing of the manuscript.

## Supporting information


**FIGURE S1** Distribution of diagnostic categories in each age range. Individuals with ASD were categorized as autism, Asperger's Disorder, Pervasive Developmental Disorder Not‐Otherwise‐Specified (PDD‐NOS), and un‐categorized subjects (unknown).
**FIGURE S2**. The small‐worldness of networks of CT/SA covariance in patients with ASD and the TDCs, respectively. Networks of SA covariance showed lower small‐worldness than that of CT covariance in all three age baskets.
**FIGURE S3**. Comparison of properties of CT network between patients with ASD and the TDCs with varying connective sparsities, in different age bands. The gray shade shows the 95% confidence interval obtained from 5,000 permutation tests, and the group differences were presented in orange dots at varying network sparsities. No significant difference was found in clustering coefficient, global efficiency, small‐worldness, or modularity between patients with ASD and the TDCs (*qs* > 0.05, permutation test, FDR corrected).
**FIGURE S4.** Whole‐brain averaged SA and CT across acquisition sites, in their initial scale (**A**), after regressing out site information (**B**), and after MAD rescaling (**C**).
**FIGURE S5.** Comparison of properties of SA and CT networks using regular permutation strategy. The gray shade shows the 95% confidence interval obtained from 5,000 permutation tests, and the group differences are presented in orange dots at varying network sparsities. For the SA network, small‐worldness of children with ASD significantly decreased at link sparsity of 15% (red arrows, *q* < 0.05, FDR corrected). No significant difference was found in clustering coefficient, global efficiency, small‐worldness, or modularity in CT network between patients with ASD and the TDCs (*qs* > 0.05, FDR corrected).
**FIGURE S6.** Comparison of different permutation strategies by recursively removing the 5 centers with the largest sample size. The permutation test was performed on the averages of clustering coefficient, shortest path length, and small‐worldness over link density of 5–35% (with 5% increment). Shaded gray areas are the 95% confidence interval obtained from 5,000 permutation tests, and the group differences are presented in orange lines. For each panel, the first row shows the comparison results from our paired permutation strategy and the second row is the results from the regular permutation strategy. Both of the two permutation strategies received similar comparison results in clustering coefficient and shortest path length. However, the results of small‐worldness showed large fluctuation when using the regular permutation method, which showed significant higher small‐worldness in children and adults with ASD relative to the matched TDCs after removing samples of the top 5 centers; whereas, no significant between‐group difference was found in small‐worldness of these two age groups using our permutation strategy.
**FIGURE S7.** The percentage changes of nodal eigenvector centrality in the ASD cohort compared to the TDCs.
**TABLE S1.** Diagnostic criteria for ASD and TDC at different contributing centers.

## Data Availability

We thank the numerous contributors to the ABIDE database for their effort in the collection, organization, and sharing of their datasets. The data that support the findings of this study are openly available at http://fcon_1000.projects.nitrc.org/indi/abide/.
