# Supplementary material for: Developmental pattern of the cortical topology in high‐functioning individuals with autism spectrum disorder
Source: Hum Brain Mapp. 2020 Oct 21;42(3):660–75. doi: 10.1002/hbm.25251 (PMC7814766; doi:10.1002/hbm.25251)
Supplement: Supplementary file 1 — FIGURE S1 Distribution of diagnostic categories in each age range. Individuals with ASD were categorized as autism, Asperger's Disorder, Pervasive Developmental Disorder Not‐Otherwise‐Specified (PDD‐NOS), and un‐categorized subjects (unknown). FIGURE S2. The small‐worldness of networks of CT/SA covariance in patients with ASD and the TDCs, respectively. Networks of SA covariance showed lower small‐worldness than that of CT covariance in all three age baskets. FIGURE S3. Comparison of properties of CT network between patients with ASD and the TDCs with varying connective sparsities, in different age bands. The gray shade shows the 95% confidence interval obtained from 5,000 permutation tests, and the group differences were presented in orange dots at varying network sparsities. No significant difference was found in clustering coefficient, global efficiency, small‐worldness, or modularity between patients with ASD and the TDCs (qs > 0.05, permutation test, FDR corrected). FIGURE S4. Whole‐brain averaged SA and CT across acquisition sites, in their initial scale (A), after regressing out site information (B), and after MAD rescaling (C). FIGURE S5. Comparison of properties of SA and CT networks using regular permutation strategy. The gray shade shows the 95% confidence interval obtained from 5,000 permutation tests, and the group differences are presented in orange dots at varying network sparsities. For the SA network, small‐worldness of children with ASD significantly decreased at link sparsity of 15% (red arrows, q < 0.05, FDR corrected). No significant difference was found in clustering coefficient, global efficiency, small‐worldness, or modularity in CT network between patients with ASD and the TDCs (qs > 0.05, FDR corrected). FIGURE S6. Comparison of different permutation strategies by recursively removing the 5 centers with the largest sample size. The permutation test was performed on the averages of clustering coefficient, shortest path length, and small‐worl [file HBM-42-660-s001.docx]

**Supplementary Materials**

**
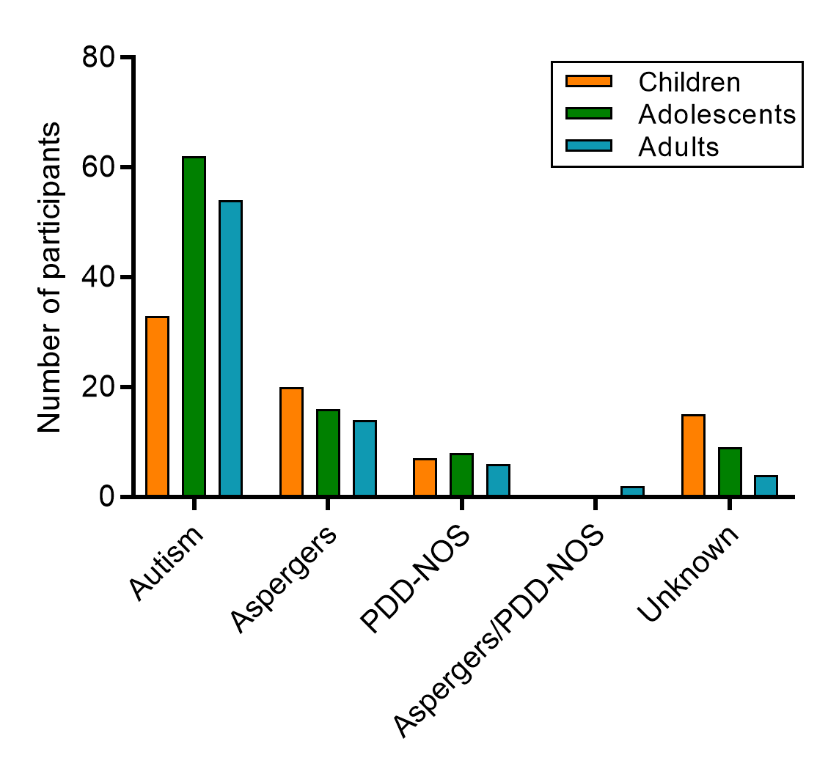
**

**Figure S1.** Distribution of diagnostic categories in each age range. Individuals with ASD were categorized as autism, Asperger's Disorder, Pervasive Developmental Disorder Not-Otherwise-Specified (PDD-NOS), and un-categorized subjects (unknown).

**
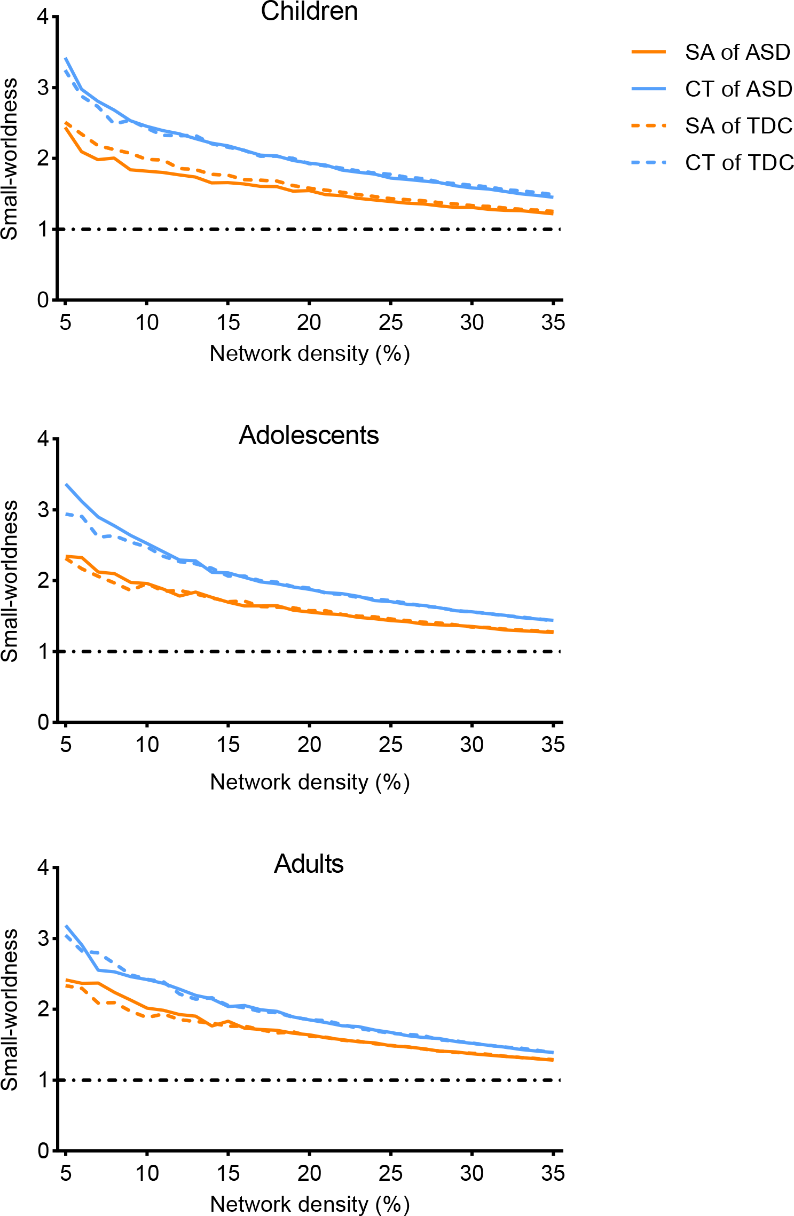
**

**Figure S2**. The small-worldness of networks of CT/SA covariance in patients with ASD and the TDCs, respectively. Networks of SA covariance showed lower small-worldness than that of CT covariance in all three age baskets.

**
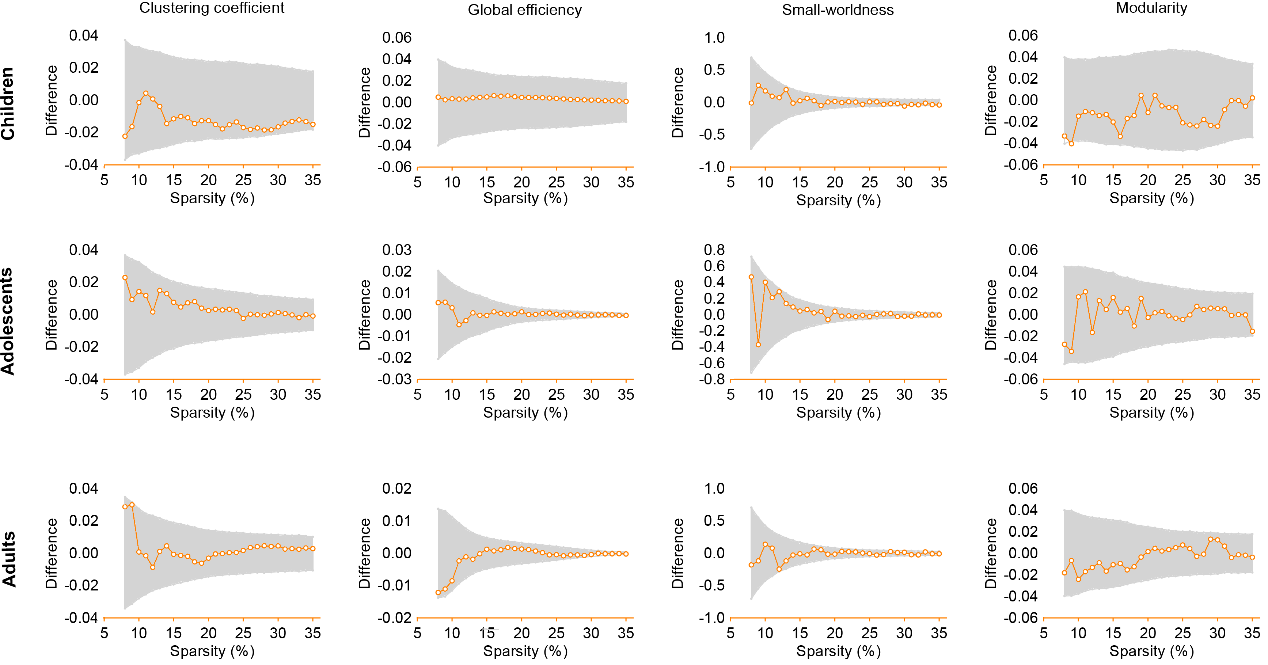
**

**Figure S3**. Comparison of properties of CT network between patients with ASD and the TDCs with varying connective sparsities, in different age bands. The gray shade shows the 95% confidence interval obtained from 5000 permutation tests, and the group differences were presented in orange dots at varying network sparsities. No significant difference was found in clustering coefficient, global efficiency, small-worldness, or modularity between patients with ASD and the TDCs (*qs* > 0.05, permutation test, FDR corrected).


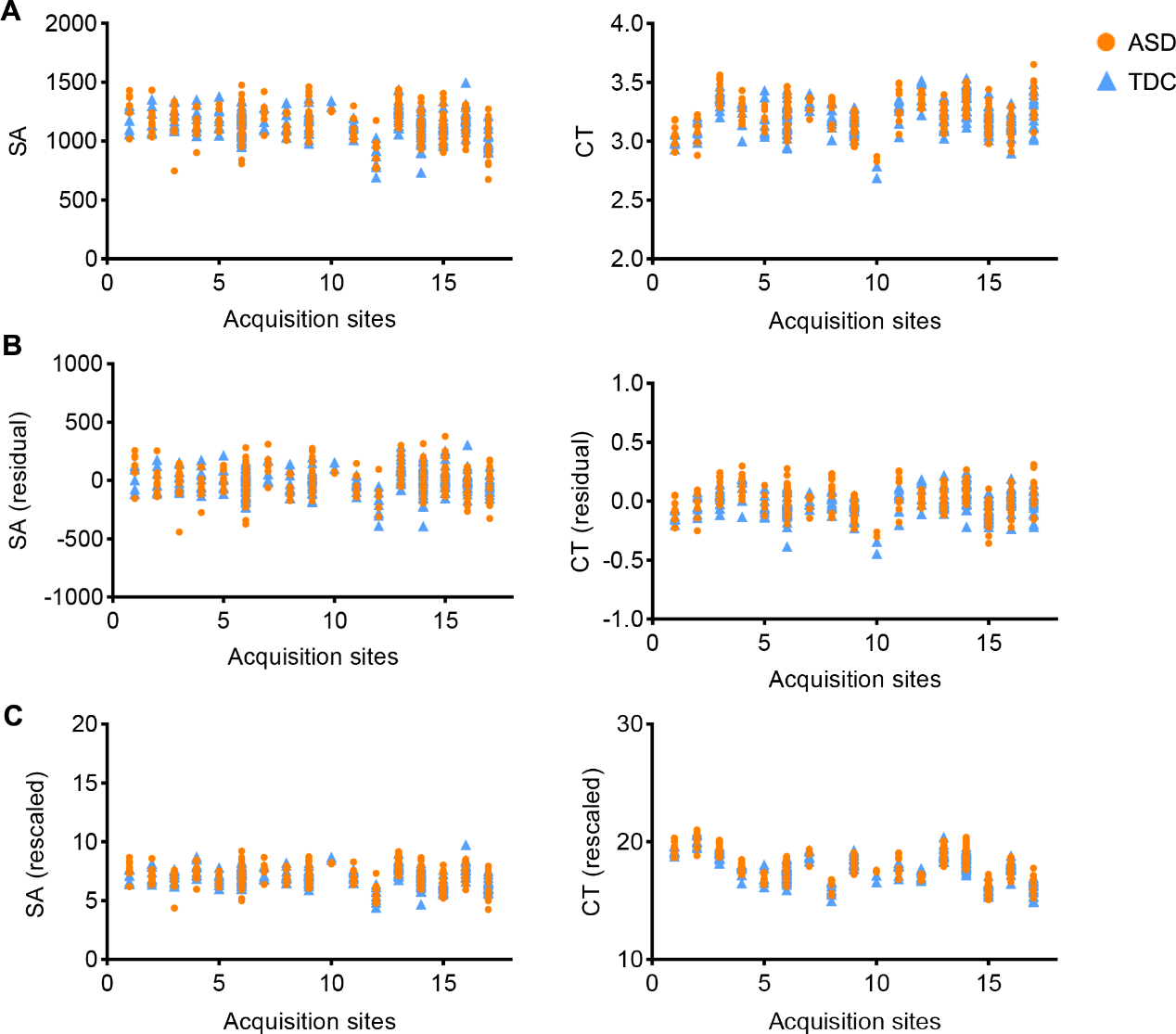


**Figure S4.** Whole-brain averaged SA and CT across acquisition sites, in their initial scale (**A**), after regressing out site information (**B**), and after MAD rescaling (**C**).


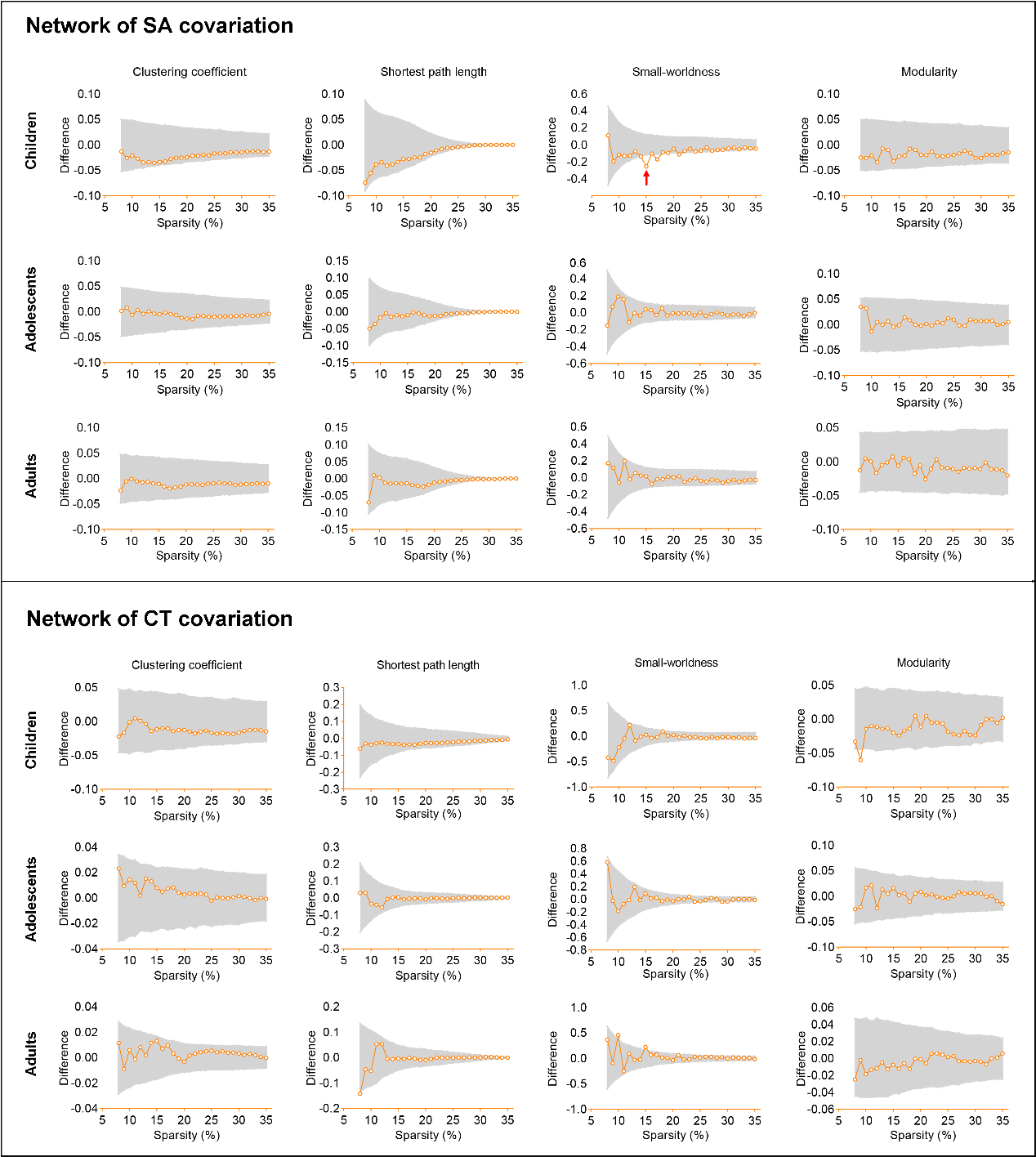


**Figure S5.** Comparison of properties of SA and CT networks using regular permutation strategy. The gray shade shows the 95% confidence interval obtained from 5000 permutation tests, and the group differences are presented in orange dots at varying network sparsities. For the SA network, small-worldness of children with ASD significantly decreased at link sparsity of 15% (red arrows, *q* < 0.05, FDR corrected). No significant difference was found in clustering coefficient, global efficiency, small-worldness, or modularity in CT network between patients with ASD and the TDCs (*qs* > 0.05, FDR corrected).


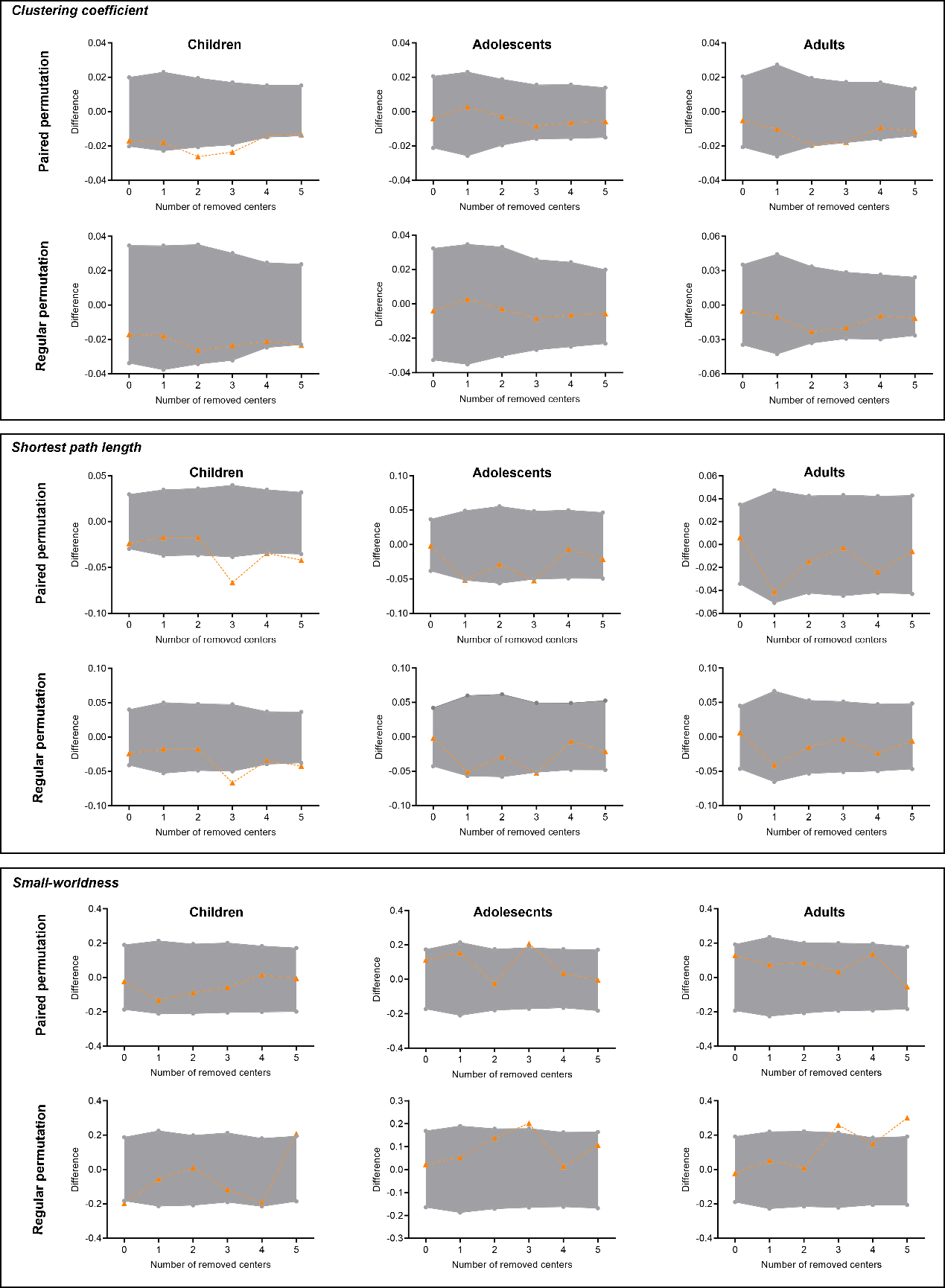


**Figure S6.** Comparison of different permutation strategies by recursively removing the 5 centers with the largest sample size. The permutation test was performed on the averages of clustering coefficient, shortest path length, and small-worldness over link density of 5% to 35% (with 5% increment). Shaded grey areas are the 95% confidence interval obtained from 5000 permutation tests, and the group differences are presented in orange lines. For each panel, the first row shows the comparison results from our paired permutation strategy and the second row is the results from the regular permutation strategy. Both of the two permutation strategies received similar comparison results in clustering coefficient and shortest path length. However, the results of small-worldness showed large fluctuation when using the regular permutation method, which showed significant higher small-worldness in children and adults with ASD relative to the matched TDCs after removing samples of the top 5 centers; whereas, no significant between-group difference was found in small-worldness of these two age groups using our permutation strategy.


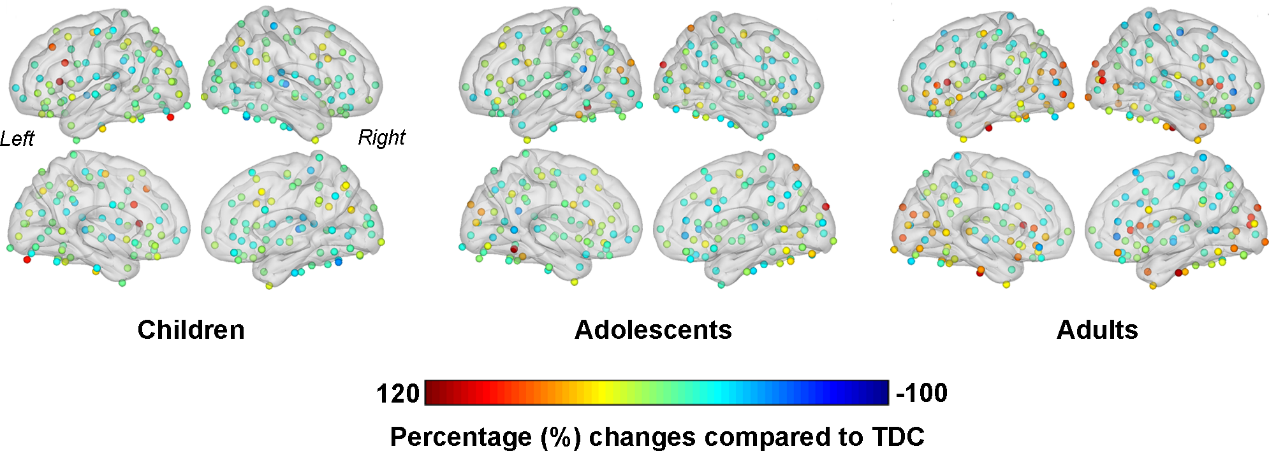


**Figure S7.** The percentage changes of nodal eigenvector centrality in the ASD cohort compared to the TDCs.

**Table S1. Diagnostic criteria for ASD and TDC at different contributing centers.**

| Acquisition senter | Scanner | Diagnostic criteria |
| --- | --- | --- |
| CALTECH | SIEMENS Trio | **ASD**   - Diagnosed based on DSM-IV-TR, ADOS, and ADI-R. - Full-scale IQ > 80. - Free of psychiatric or neurological conditions, history of epilepsy, or brain injury.   **TDC**   - Matched at the group level to ASD relative to age, sex, handedness, and full-scale IQ. - No family history of an ASD. - No history of ASD or any psychiatric or neurological condition. |
| CMU | SIEMENS Verio | **ASD**   - Diagnosed based on ADOS, ADI-R, and expert clinical diagnosis. - Medically healthy and had no identifiable genetic, metabolic, or infectious etiology for their disorder. - No psychiatric or neurological conditions, history of epilepsy, clinically active seizures, or brain injury.   **TDC**   - No family history of an ASD. - No history of ASD or any psychiatric or neurological condition. |
| KKI | Philips Achieva | **ASD**   - Diagnosed based on DSM-IV-TR, ADOS-G, and ADI-R. - Participants were screened for other psychiatric diagnoses by the DICA-IV.   **TDC**   - Free of psychiatric or neurological conditions screened by the DICA-IV. - No family history of ASD. |
| LEUVEN1 | Philips INTERA | **ASD**   - Diagnosed based on DSM-IV-TR and Autism Quotient (AQ). - Social Responsiveness scale (SRS) > 60. - Free of psychiatric or neurological conditions, history of epilepsy, and brain injury.   **TDC**   - Matched at the group level to ASD relative to age, sex, handedness, and full-scale IQ. - No history of psychiatric or neurological condition. |
| MAX_MUM | SIEMENS Verio | **ASD**   - Diagnosed based on ADI-R, ADOS, and AQ. - Excluded individuals with secondary autism related to a specific etiology, such as tuberous sclerosis or Fragile X syndrome. - Free of psychiatric and neurological conditionsm, seizure, head injury, toxic exposure and the evidence of genetic, metabolic, or infectious disorders.   **TDC**   - Matched at the group level to ASD relative to age. - Absence of reported history of major psychiatric disorder, seizure, head injury, or toxic exposure; no evidence of genetic, metabolic, or infectious disorders. - No history of delayed language development. |
| NYU | SIEMENS Allegra | **ASD**   - Diagnosed based on DSM-IV-TR, ADOS, and ADI-R. - Parent interview using the Schedule of Affective Disorders and Schizophrenia for Children-Present and Lifetime Version (KSADS-PL) for children. - Participant interview using the Structured Clinical Interview for DSM-IV-TR Axis-I Disorders, Non-patient Edition (SCID-I/NP) and the Adult ADHD Clinical Diagnostic Scale (ACDS) for adults. - Free of manic or depressive episode, bipolar disorder, schizophrenia, or posttraumatic stress disorder.   **TDC**   - Matched at the group level to ASD relative to age and sex. - Absence of any current Axis-I disorders based on the KSADS-PL (for children and their parents), and SCID-I/NP and ACDS interviews(for adults). |
| OHSU | SIEMENS Trio | **ASD**   - Diagnosed based on ADOS, ADI-R, and clinical interview. - Full-scale IQ > 80. - Free of psychiatric or neurological conditions, and conduct disorder. - Parents had no history of neurological illness, chronic medical problems, sensorimotor handicap, intellectual disability, significant head trauma (with loss of consciousness).   **TDC**   - Absence of any disorders based on Kiddie Schedule for Affective Disorders and Schizophrenia (K-SADS-E), parent Conners' Rating Scale-3rd Edition, ADHD Rating Scale and Strengths and Difficulties Questionnaire, and a clinical review by a child psychiatrist and/or a neuropsychologist. - Full-scale IQ > 80. - Free of psychiatric or neurological conditions, and conduct disorder. - Parents had no history of neurological illness, chronic medical problems, sensorimotor handicap, intellectual disability, significant head trauma (with loss of consciousness). |
| OLIN | SIEMENS Allegra | **ASD**   - Diagnosed based on ADOS, ADI-R, SRS, and the Social Communication Questionnaire (SCQ)-Lifetime form. - Full-scale IQ > 70.   **TDC**   - ASD was ruled out in TDC using a detailed health questionnaire. - Evaluated based on the ADOS or the SCQ-Lifetime form (scores were below diagnostic curoff value). |
| PITT | SIEMENS Allegra | **ASD**   - Diagnosed based on ADOS-G, ADI-R, and clinical interview. - Full-scale IQ > 80. - No history of head injury (with loss of consciousness), Epilepsy, Meningitis, Encephalitis, tuberous sclerosis or Fragile X syndrome; no genetic, psychiatric or neurological disorder. - Participants with PDD-NOS or Asperger's syndrome (i.e., no language delay evident) were excluded   **TDC**   - Matched individually to the ASD participants on age, full-scale IQ, and gender. - No history of head trauma, birth complications, seizures; no psychiatric, genetic or neurological disorder. |
| SBL | Philips INTERA | **ASD**   - Diagnosed based on DSM-IV-TR and AQ, and a part of participants were also assessed by ADOS. - IQ in the normal range. - Free of psychiatric (assessed using the Dutch version of the SCAN 2.1) or neurological conditions, history of epilepsy, or brain injury.   **TDC**   - Matched at the group level to ASD relative to age. - IQ in the normal range. - Free of psychiatric or neurological conditions, history of epilepsy, or brain injury. |
| SDSU | GE MR750 | **ASD**   - Diagnosed based on DSM-IV-TR, ADOS, and ADI-R. - No ASD-related medical conditions (e.g., Fragile-X syndrome, tuberous sclerosis), and other neurological conditions (e.g., epilepsy, Tourette's Syndrome).   **TDC**   - No personal or family history of ASD; no reported history of any other neurological or psychiatric conditions. - Met the criteria using a questionnaire developed within the lab. |
| STANFORD | GE Signa | **ASD**   - Diagnosed based on ADOS and ADI-R. - Full-scale IQ > 70. - No history of known genetic, psychiatric, or neurological disorders (e.g., Fragile X syndrome or Tourette's syndrome); or no antipsychotic medication usage.   **TDC**   - Full-scale IQ > 70. - Participants or a first-degree relative had no developmental, language, learning, neurological, psychiatric disorders, or psychiatric medication usage. - Participants who met the Child Symptom Inventory-Fourth Edition or Child and Adolescent Symptom Inventory were excluded. |
| TRINITY | Philips Achieva | **ASD**   - Diagnosed based on DSM-IV-TR, ADOS, and ADI-R. - Righted handed male with full-scale IQ > 70. - No history of neurological, psychiatric, or genetic disorder (e.g. Anxiety, Depression, a psychotic disorder, Obesessive Compulsive Disorder, Epilepsy/seizures, Fragile X syndrome, Tourettes Syndrome, or Tuberous Sclerosis). - No usage of psychotropic medication.   **TDC**   - Matched at the group level to ASD relative to age, gender, handedness, and full-sacle IQ. - Absence of any psychiatric, neurological and genetic disorders including dyslexia or dyspraxia. - No first degree relative who has been diagnosed with ASD. - Raw score < 50 on the SRS-Child version and < 10 on the Social Communication Questionnaire (SCQ). |
| UCLA1 & 2 | SIEMENS Trio | **ASD**   - Diagnosed based on ADOS and ADI-R. - No history of seizures, no loss of consciousness for more than 5 minutes, no tic disorder or involuntary movements, no any known genetic, neurological, or psychiatric disorder.   **TDC**   - No history of any genetic, neurological, psychiatric, or developmental disorders. - Participants had no first degree relative with an ASD diagnosis. |
| UM1 | GE Signa | **ASD**   - Diagnosed based on ADOS-G, ADI-R, and clinical interview. - Age > 7, non-verbal IQ ≥ 85. - No history of head trauma, neurological (including seizures), or psychiatric disorder. - No usage of psychotropic medications.   **TDC**   - Age > 7, non-verbal IQ ≥ 85. - No history of head trauma, neurological (including seizures), or psychiatric disorder. - Received a score < 10 on the SCQ and a score < 6 on the Obsessive/Compulsive subscale of the Spence Children's Anxiety scale (SCAS). |
| UM2 | GE Signa | **ASD**   - Diagnosed based on ADOS-G, ADI-R, and clinical interview. - Age > 13, non-verbal IQ ≥ 80. - No history of head trauma, psychosis, bipolar disorder, or a neurological disorder (including seizures) .   **TDC**   - Age > 13, non-verbal IQ ≥ 80. - No history of head trauma, psychosis, bipolar disorder, or a neurological disorder (including seizures) . - Received a score < 10 on the SCQ and a score < 6 on the SCAS. |
| USM | SIEMENS Trio | **ASD**   - Diagnosed based on DSM-IV-TR, ADOS, and ADI-R. - Full-scale IQ > 70. - Absence of known medical causes of autism (such as tuberous sclerosis, Fragile X, or neonatal ischemic/hypoxia); no blindness or deafness; no history of seizures, severe head injury, or severe medical problems.   **TDC**   - Matched to the ASD group relative to age. - Full-scale IQ > 70. - No history of learning disabilities, neurological disorder, severe head injury, neonatal ischemia/hypoxia, substance abuse, psychiatric disorder, or family history of ASD in 1st, 2nd, or 3rd degree relatives. |
| Yale | SIEMENS Trio | **ASD**   - Diagnosed based on DSM-IV-TR, ADOS, and ADI-R. - Free of psychiatric or neurological conditions, history of epilepsy, or brain injury.   **TDC**   - Free of neuropsychiatric or neurodevelopmental disorders as determined via parent report.   **Other exclusion criteria to all participants**   - All participants had normal or corrected to normal vision. All participants spoke English as their primary language in the home. |

CALTECH, California Institute of Technology; CMU, Carnegie Mellon University; KKI, Kennedy Krieger Institute; LEUVEN, University of Leuven; MAX_MUM, Ludwig Maximilians University Munich; NYU, New York University Langone Medical Center; OHSU, Oregon Health and Science University; OLIN, Olin Center, Institute of Living at Hartford Hospital; PITT, University of Pittsburgh; SBL, Social Brain Lab, BCN Neuroimaging Center, University Medical Center Groningen; SDSU, San Diego State University; STANFORD, Stanford University; TRINITY, Trinity Center for Health Sciences; UCLA, University of California, Los Angeles; UM, University of Michigan; USM, University of Utah; Yale, Yale Child Study Center.
